# Supplementary material for: Characterization and Comparative Analysis of Chloroplast Genomes of Medicinal Herb Scrophularia ningpoensis and Its Common Adulterants (Scrophulariaceae)
Source: Int J Mol Sci. 2023 Jun 12;24(12):10034. doi: 10.3390/ijms241210034 (PMC10298345; doi:10.3390/ijms241210034)
Supplement: Supplementary file 1 [file ijms-24-10034-s001.zip › Supplementary Figure.pdf]

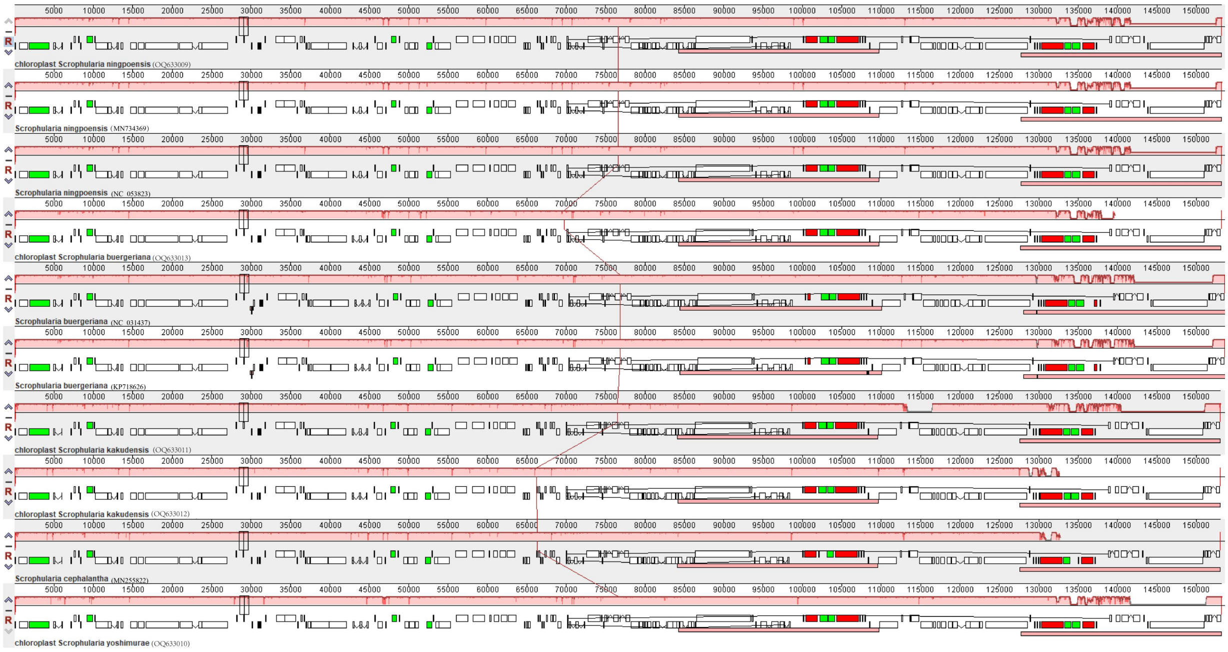

Supplementary Figure S1. The collinearity analysis diagram of 10 *Scrophularia* individuals.

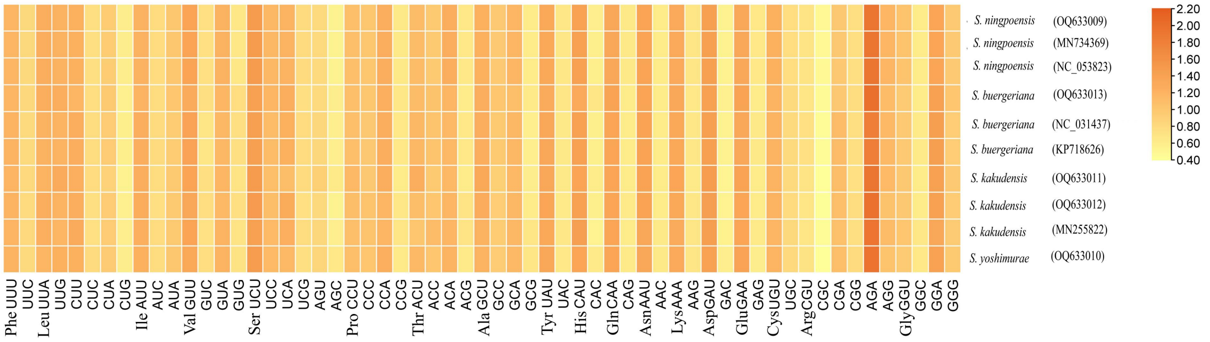

Supplementary Figure S2. Codon bias heat map of 10 *Scrophularia* individuals.
