# Supplementary material for: Characterization and Comparative Analysis of Chloroplast Genomes of Medicinal Herb Scrophularia ningpoensis and Its Common Adulterants (Scrophulariaceae)
Source: Int J Mol Sci. 2023 Jun 12;24(12):10034. doi: 10.3390/ijms241210034 (PMC10298345; doi:10.3390/ijms241210034)
Supplement: Supplementary file 1 [file ijms-24-10034-s001.zip › Table S2.pdf]

**Supplementary Table S2.** Gene composition of *Scrophularia* chloroplast genomes.

| Groups of Gene | Name of Gene |
|---|---|
| Ribosomal RNAs | *rrn16(×2), rrn23(×2), rrn4.5(×2), rrn5(×2)* |
| Transfer RNAs | [a] *trnA-UGC(×2), trnC-GCA, trnD-GUC, trnE-UUC, trnF-GAA, trnfM-CAU,* [a] *trnG-GCC trnG-UCC, trnI-CAU(×2), trnH-GUG,* [a] *trnI-GAU(×2), trnK-UUU, trnL-CAA(×2)* [a] *trnL-UAA, trnL-UAG, trnM-CAU, trnN-GUU(×2), trnP-UGG, trnQ-UUG, trnR-ACG(×2) trnR-UCU, trnS-GCU, trnS-GGA, trnS-UGA, trnT-GGU, trnT-UGU, trnV-GAC(×2),* [a] *trnV-UAC, trnW-CCA, trnY-GUA* |
| Photosystem I | *psaA, psaB, psaC, psaI, psaJ* |
| Photosystem II | *psbA, psbB, psbC, psbD, psbE, psbF, psbH, psbI, psbJ, psbK, psbL, psbM, psbN, psbT, psbZ* |
| Cytochrome | *petA,* [a] *petB,* [a] *petD, petG, petL, petN* |
| ATP synthase | *atpA, atpB, atpE,* [a] *atpF, atpH, atpI* |
| Rubisco | *rbcL* |
| NADH dehydrogenase | *ndhA,* [a] *ndhB(×2), ndhC, ndhD, ndhE, ndhF, ndhG, ndhH, ndhI, ndhJ, ndhK* |
| ATP-dependent protease subunit P | [b] *clpP* |
| Chloroplast translational initiation factor | *infA* |
| Chloroplast envelope membrane protein | *cemA* |
| Large units | *rpl33, rpl20, rpl36, rpl14,* [a] *rpl16,* [a] *rpl2(×2), rpl23(×2), rpl32, rpl22* |
| Small units | [a] *rps16, rps2, rps14, rps4, rps18,* [b] *rps12(×2), rps11, rps8, rps19, rps3, rps7(×2), rps15* |
| RNA polymerase | *rpoC2,* [a] *rpoC1, rpoB, rpoA* |
| Miscellaneous proteins | *matK, accD, ccsA* |
| Hypothetical proteins and conserved reading frame | [b] *ycf3, ycf4, ycf2(×2), ypcf1, ycf15(×2)* |

a Indicates the genes containing a single intron. b Indicates the genes containing two introns. (×2) Indicates genes duplicated in the IR regions.
