# Supplementary material for: Characterization and Comparative Analysis of Chloroplast Genomes of Medicinal Herb Scrophularia ningpoensis and Its Common Adulterants (Scrophulariaceae)
Source: Int J Mol Sci. 2023 Jun 12;24(12):10034. doi: 10.3390/ijms241210034 (PMC10298345; doi:10.3390/ijms241210034)
Supplement: Supplementary file 1 [file ijms-24-10034-s001.zip › Table S8.pdf]

Table S8. The list of 28 chloroplast genomes from Scrophulariaceae in phylogenetic analyses.

**Genus**	**Taxon**	**GenBank Accession**
Scrophularia	*S.buergeriana*	KP718626
	S.buergeriana	NC_031437
	S.buergeriana	OQ633013
	S.yoshimurae	OQ633010
	S.ningpoensis	MN734369
	S.ningpoensis	NC_053823
	S.ningpoensis	OQ633009
	S.kakudensis	OQ633012
	S.kakudensis	MN255822
	S.kakudensis	OQ633011
	S. henryi	MF861203
	S. takesimensis	KP718628
	S. incisa	OP036429
	S. incisa	OP018675
	S. kiriloviana	OP018676
	S. kiriloviana	OP036428
	S. dentata	MF861202
	S. dentata	OP018677
	S. integrifolia	OP018678
Verbascum	*V. chinense*	MT610040
	V. phoeniceum	MN893301
Buddleja	*B. officinalis*	MZ955034
	B. sessilisfolia	MH411149
Diocirea	*D. violacea*	MN044644
Eremophila	*E. oppositifolia*	MN044645
Myoporum	*M. bontioides*	NC050956
	M. laetum	MN044641
Leucophyllum	*L. frutescens*	MN044638
